# Predialysis Nephrology Care Disparities and Incident Vascular Access Among Hispanic Individuals

**DOI:** 10.1001/jamanetworkopen.2025.30972

**Published:** 2025-09-05

**Authors:** Sheena Pramod, Grant Scheiffele, Wenxi Huang, Cydney Parmar, Yi Guo, Jiang Bian, Serena Jingchuan Guo, Ashutosh M. Shukla

**Affiliations:** 1 Division of Nephrology, Hypertension, and Transplantation, Department of Medicine, University of Florida, Gainesville; 2 North Florida/South Georgia Veterans Health System, Gainesville, Florida; 3 Department of Health Outcomes & Biomedical Informatics, University of Florida, Gainesville; 4 Department of Pharmaceutical Outcomes and Policy, University of Florida, Gainesville

## Abstract

**Importance:**

Predialysis nephrology care is associated with the likelihood of having a mature, usable arteriovenous access for starting hemodialysis (ie, incident vascular access), a key care quality metric for patients with kidney failure. However, the magnitude of this association has not been quantified to date.

**Objective:**

To quantify the attributable association between lack of access to predialysis nephrology care and incident vascular access outcomes among Hispanic patients.

**Design, Setting, and Participants:**

This cohort study is a retrospective analysis of the 2021 US Renal Database System. Participants were all adult Medicare recipients initiating hemodialysis between 2010 and 2019; primary analysis was restricted to those with at least 6 months of predialysis Medicare status. Data analysis was performed from June 2022 to November 2024.

**Exposure:**

Self-reported race and ethnicity, with the non-Hispanic White category serving as the reference and Hispanic ethnicity as the primary comparator. Any predialysis nephrology care was the primary mediator, and at least 6 months of nephrology care and predialysis kidney disease education were the mediators for sensitivity analyses.

**Main Outcomes and Measures:**

The attributable association between predialysis nephrology care and incident vascular access (ie, the composite of arteriovenous fistula [AVF] or arteriovenous graft [AVG]) disparity was the primary outcome, and its attributable association between remaining incident access types, including central venous catheter (CVC) with maturing in-situ AVF or AVG, and CVC without any other access (CVC only) disparity, were the secondary outcomes. Causal mediation analysis with logistic regression was used to determine the unadjusted and adjusted associations.

**Results:**

Among 427 340 eligible patients undergoing incident hemodialysis (mean [SD] age, 72.65 [10.68] years; 241 420 male [56.5%]), 92 887 (21.7%) were Black, 46 146 (10.8%) were Hispanic, 269 697 (63.1%) were White, and 18 610 (4.35%) were other races and ethnicities. AVF was used in 62 075 patients (14.5%), AVG in 13 163 patients (3.1%), and CVC in 351 315 patients (82.2%). Compared with White patients, Hispanic patients had adjusted odds ratios (aORs) of 0.70 (95% CI, 0.68-0.72) for receiving predialysis nephrology care and 0.77 (95% CI, 0.75-0.80) for receiving incident vascular access, for a 23% lower rate. A lack of nephrology care accounted for 32.59% of incident vascular access and 62.00% of maturing vascular access underuse. Sensitivity analyses enhancing the predialysis care disparities strengthened incident vascular access disparity and the attributable association. Secondary analyses revealed that compared with White patients, Hispanic individuals with CVC and a maturing AVF or AVG had 38% (aOR, 1.38; 95% CI, 1.23-1.53) higher odds and those with CVC only had 30% (aOR, 1.30; 95% CI, 1.25-1.35) higher odds of conversion to a functional AVF or AVG within the first year of dialysis, with predialysis care negatively mediating these outcomes.

**Conclusions and Relevance:**

This retrospective cohort study of incident hemodialysis patients found that system-based disparities in predialysis access to nephrology care contribute to approximately one-third of incident vascular access disparities among Hispanic individuals. Targeted system-based remedies and policies are needed to improve timely identification and nephrology referrals among Hispanic individuals, for equitable improvements in incident kidney failure outcomes.

## Key Points

**Question:**

Are disparities in predialysis care associated with having a mature, usable vascular access at dialysis initiation among Hispanic individuals?

**Findings:**

In this cohort study of 427 340 eligible patients using data from the US Renal Data System, Hispanic individuals had 23% lower rates of mature usable vascular access at dialysis initiation vs White individuals, despite being younger and having less comorbidity burden. Approximately one-third (32.59%) of these differences were attributable to a lack of access to nephrology care before dialysis.

**Meaning:**

These findings suggest that targeted system-based remedies and policies are required to improve timely identification and nephrology referrals among Hispanic individuals to achieve equitable improvements in incident kidney failure outcomes.

## Teaser

This cohort study examines the association of lack of access to predialysis nephrology care with incident vascular access outcomes among Hispanic patients.

## Introduction

Hemodialysis is the dominant kidney replacement therapy (KRT) and is used in approximately 85% of patients initiating dialysis in the US.^1^ Compared with a central venous catheter (CVC), performing hemodialysis with an arteriovenous vascular access (ie, arteriovenous fistula [AVF] or arteriovenous graft [AVG]) is associated with reduced infections, hospitalizations, and health care costs, with a few studies showing improved survival.^2^ Consequently, regulatory bodies, including the Centers for Medicare & Medicaid Services (CMS) National Quality Forum, have designated vascular access rates as a core quality metric and issued systemwide initiatives prioritizing it.^3,4^ Such enhanced regulatory focus has greatly increased the prevalent vascular access rates for US patients undergoing dialysis.^1^ However, despite nearly 2 decades of the established target of 65%, rates of incident vascular access (ie, initiating hemodialysis with either an AVF or AVG) remain suboptimal.^1^ Currently, less than 20% of all patients initiating hemodialysis in the US use AVF or AVG, with even lower rates in marginalized populations.^1^

Incident vascular access requires timely identification of advanced chronic kidney disease, referral to nephrology services, and a favorable health care infrastructure with appropriate expertise to facilitate creation and maturation of AVF or AVG.^5,6,7^ As such, system-level access to care factors (ie, presence and quality of predialysis clinical care, specifically nephrology care and KRT-directed kidney disease education [KDE]) is considered a prerequisite for improving incident vascular access outcomes.^8^

Studies show that the Hispanic population has among the lowest rates of incident vascular access outcomes across all races and ethnicities,^9,10^ with experts and a few qualitative studies indicating major role for various patient-level and practitioner-level factors.^5^ However, it is well known that Hispanic individuals have substantial disparities in predialysis clinical care, including the presence and quality of nephrology care.^11,12^ Despite the sequential nature of these inadequacies, the relative importance of these upstream advanced chronic kidney disease care disparities in driving downstream incident vascular access differences has not been elucidated. Establishing their importance across multiple potential barriers is crucial for identifying stakeholders and strategies required to address these deficits. Using the 2021 United States Renal Data System (USRDS), we aimed to evaluate the attributable association between inequalities in predialysis nephrology care and incident vascular access among Hispanic individuals.

## Methods

### Study Design

This retrospective cohort study was approved by the University of Florida institutional review board under an expedited, informed consent–exempt study, because the data were publicly available and deidentified, in accordance with 45 CFR §46. To report our findings, we followed the Strengthening the Reporting of Observational Studies in Epidemiology (STROBE) reporting guidelines for cohort studies.^13^ We used the standard files from the 2021 USRDS linked with Medicare claims data as the primary dataset to identify all adult (≥18 years old) patients with incident kidney failure with their first-ever hemodialysis between July 1, 2010, and December 31, 2019. Considering the time needed for referral, creation, and maturation of a ready-to-use vascular access at dialysis initiation, and the overriding impact of predialysis insurance or Medicare on incident vascular access outcomes,^14^ we used the treatment history files to restrict our primary analysis cohort to patients with at least 6 months of predialysis Medicare coverage. We used the USRDS end-stage kidney disease and pre–end-stage kidney disease Institutional and Physician/Supplier claim and the Healthcare Common Procedure Coding System codes of G0420 and G0421 to identify predialysis KDE service recipients, which, per regulations, included education targeting KRT modality and vascular access. We excluded patients missing the first date of dialysis or CMS Medical Evidence Report Form (CMS-2728) and receiving transplants before or KDE after initiating hemodialysis. We used CMS-2728 form and core USRDS dataset to retrieve information on patient demographics, key medical comorbidities, functional status, and laboratory data, including serum albumin.

### Exposures, Mediators, and Outcome Measures

Our causal mediation model (eFigure 1 in Supplement 1) used self-reported ethnicity and race as the exposure of interest, with Hispanic White referred to as Hispanic, Black race as Black, and non-Hispanic White as White. Predialysis nephrology care was the hypothesized mediator for the primary analysis, with increased intensity, defined as longer than 6 months of predialysis nephrology care,^15,16^ and increased quality, characterized by predialysis KDE services, as the hypothesized mediators for sensitivity analyses.^17^ Per recent guidelines, we prioritized incident vascular access, the composite of AVF or AVG use,^4,5^ as the primary outcome, and other vascular access outcomes, such as incident AVF, AVG, CVC only, and CVC with maturing AVF or AVG, as secondary outcomes.

We used the causal mediation model described by Baron et al^18^ for our mediation approach, which requires a priori establishment of significant associations in each group of the directed acyclic graphs (ie, between the exposure [race and ethnicity], mediators, and outcomes). On the basis of a prior publication^18^ and the results of our preliminary exposure to outcome assessments, the Hispanic population fulfilled the mediation model prerequisites for all primary and secondary vascular access outcomes, and thus, was targeted for primary analysis. Owing to the limited difference in composite vascular access outcomes between the White and Black individuals, we evaluated the significant disparity in incident AVF rates among Black patients for secondary analyses to confirm the directionality and strength of our primary findings. Finally, we performed a sensitivity analysis that included all patients with active Medicare status at hemodialysis initiation, as well as secondary analyses to examine incident CVC to AVF or AVG conversion during the first year of dialysis, providing indirect indications regarding the medical fitness of the populations.

### Statistical Analysis

Data analysis was performed from June 2022 to November 2024. We used SAS statistical software version 9.4 (SAS Institute) for all analyses, including causal mediation analysis (PROC CAUSALMED). Demographic, clinical, and socioeconomic characteristics were compared between the study groups using χ^2^ tests for categorical variables and the 1-way analysis of variance and the Kruskal-Wallis tests for normally and nonnormally distributed continuous variables, respectively. We used logistic regression using the complete case analysis in SAS to determine the unadjusted and adjusted associations across all 3 groups of the directed acyclic graph of the mediation model (eFigure 1 in Supplement 1), excluding individuals with missing data. The adjustment models included biological factors known to be associated with vascular access outcomes, including age, sex, diabetes, body mass index, congestive heart failure, arteriosclerotic heart disease, available marker of financial state (ie, individuals’ low-income subsidy status), and important markers of dialysis facility (ie, for-profit vs nonprofit status and rurality). We reported estimated adjusted odds ratios (aORs) and 95% CIs for the effects of exposure on outcomes by total effect, which is a product of natural direct effect, defined as the effect of race or ethnicity on vascular access that is independent of the mediator, and natural indirect effect, defined as the effect of race or ethnicity on vascular access that is due to the mediator, and derived the percentage mediated for each model individually. We accounted for interactions between the exposure (ethnicity and race) and the mediator when estimating the effects of the mediation. All statistical tests were 2-sided, and a significance threshold of *P* < .01 was applied to account for multiple comparisons across multiple outcomes.

## Results

Of the 427 340 patients with at least 6 months of active predialysis Medicare coverage (mean [SD] age, 72.65 [10.68] years; 241 420 male [56.5%]), 269 697 (63.1%) were identified as White, 46 146 (10.8%) as Hispanic, 92 887 (21.7%) as Black, and 18 610 (4.35%) as other race and ethnicity individuals, which includes American Indian or Alaska Native, Asian, Native Hawaiian or Other Pacific Islander, multiracial, or unknown (eFigure 2 in Supplement 1). Compared with White individuals, Hispanic individuals were younger, had lower employment, higher rurality, higher prevalence of diabetes, and greater probability of receiving dialysis from for-profit facilities (Table 1).

**Table 1.** Demographic and Clinical Characteristics of the Study Cohort

Characteristic	Patients, No. (%)		
	Black non-Hispanic (n = 92 887)^a^	Hispanic (n = 46 146)^a^	White non-Hispanic (n = 269 697)
Age group, y			
18-44	2924 (3.1)	883 (1.9)	3293 (1.2)
45-64	22 623 (24.4)	9375 (20.3)	39 579 (14.7)
>65	67 340 (72.5)	35 888 (77.8)	226 825 (84.1)
Sex			
Female	47 522 (51.2)	19 624 (42.5)	110 152 (40.8)
Male	45 365 (48.8)	26 522 (57.5)	159 545 (59.2)
Employment status			
Employed	1765 (1.9)	751 (1.6)	6828 (2.5)
Retired	72 046 (77.6)	35 137 (76.1)	230 584 (85.5)
Unemployed or other	19 076 (20.5)	10 258 (22.2)	32 285 (12.0)
Primary disease			
Diabetes	43 936 (47.3)	30 550 (66.2)	120 555 (44.7)
Hypertension	36 185 (39.0)	10 059 (21.8)	84 530 (31.3)
Glomerulonephritis	2754 (3.0)	1259 (2.7)	12 373 (4.6)
Cystic kidney disease	487 (0.5)	174 (0.4)	2496 (0.9)
Other urologic diseases	599 (0.6)	403 (0.9)	4756 (1.8)
Other causes	7185 (7.7)	3128 (6.8)	37 906 (14.1)
Unknown cause	1740 (1.9)	573 (1.2)	7074 (2.6)
Comorbidities			
Diabetes	62 348 (67.1)	36 565 (79.2)	165 759 (61.5)
Congestive heart failure	36 025 (38.8)	17 131 (37.1)	114 001 (42.3)
Hypertension	83 668 (90.1)	41 021 (88.9)	230 563 (85.5)
Chronic obstructive pulmonary disease	10 658 (11.5)	3607 (7.8)	47 150 (17.5)
Peripheral vascular disease	11 593 (12.5)	8363 (18.1)	43 798 (16.2)
Coronary artery disease	14 651 (15.8)	10 341 (22.4)	66 305 (24.6)
Cerebrovascular disease	13 251 (14.30)	5051 (10.9)	29 581 (11.0)
Albumin, mean (SD), g/dL	3.09 (1.47)	3.07 (3.19)	3.15 (3.66)
Body mass index >30^b^	34 545 (37.2)	15 046 (32.6)	104 112 (38.6)
Needs assistance	19 487 (21.0)	10 647 (23.1)	52 984 (19.6)
Inability to ambulate	11 918 (12.8)	5969 (12.9)	30 017 (11.1)
Facility for-profit status			
For profit facility	70 996 (76.4)	37 493 (81.2)	206 631 (76.6)
Nonprofit facility	5572 (6.0)	1664 (3.6)	19 721 (7.3)
Unknown	16 349 (17.6)	6989 (15.1)	43 435 (16.1)
Low-income subsidy recipients	61 145 (65.8)	32 631 (70.7)	164 348 (60.9)
Rurality			
Metropolitan	75 145 (80.9)	34 719 (75.2)	186 483 (69.1)
Micropolitan	6874 (7.4)	2498 (5.4)	32 418 (12.0)
Rural	5027 (5.4)	1405 (3.0)	26 146 (9.7)
Missing	5841 (6.3)	7524 (16.3)	24 650 (9.1)
Predialysis nephrology care			
No nephrology care	40 886 (44.0)	20 764 (45.0)	97 072 (36.0)
Any nephrology care	52 001 (56.0)	25 382 (55.0)	172 625 (64.0)
>6 mo Nephrology care	39 112 (42.1)	18 890 (40.9)	134 135 (49.7)
Predialysis kidney replacement therapy–directed education	1180 (1.3)	412 (0.9)	4030 (1.5)
Incident access modality			
Incident composite AVF or AVG	16 179 (17.4)	6625 (14.4)	48 887 (18.1)
Incident AVF	11 649 (12.5)	5608 (12.2)	41 946 (15.6)
Incident AVG	4530 (4.9)	1017 (2.2)	6941 (2.6)
Incident maturing AVF or AVG	17 084 (18.4)	8198 (17.8)	45 852 (17.0)
Incident central venous catheter only	59 475 (64.0)	31 240 (67.7)	174 442 (64.7)

Abbreviations: AVF, arteriovenous fistula; AVG, arteriovenous graft.

SI conversion factor: To convert albumin to grams per liter, multiply by 10.

^a^ All parameters within the cohort except incident access modality were found to be statistically significantly different with *P* < .001 vs the non-Hispanic White individuals. The comparison for the incident access modality is available in Table 2.

^b^ Body mass index is calculated as weight in kilograms divided by height in meters squared.

A total of 261 527 patients (61.1%) had nephrology care and 165 813 (38.9%) did not have such care; 5895 patients (1.4%) had KDE and 421 445 (98.6%) did not. The odds of predialysis nephrology care were significantly lower for Hispanic (aOR, 0.70; 95% CI, 0.68-0.72) and Black (aOR, 0.79; 95% CI, 0.77-0.80) individuals than for White individuals (Table 2). Similar directions and larger aORs for disparity were present for a longer duration of predialysis nephrology care and KDE services. Examining incident vascular access, 351 315 patients (82.2%) used CVC, 62 075 (14.5%) used AVF, and 13 163 (3.1%) used AVG, leaving 75 238 (17.6%) who used AVF or AVG access. Compared with White individuals, Hispanic individuals had significant disparities in all incident vascular access types, with a complementary higher use of incident CVC (aOR, 1.17; 95% CI, 1.14-1.20; *P* < .001). In comparison, Black individuals only had lower odds of incident AVF (aOR, 0.85; 95% CI, 0.83-0.87; *P* < .001) but did not have disparities in other vascular access outcomes, with incident AVG rates significantly higher than those for White individuals (aOR, 1.87; 95% CI, 1.79-1.96; *P* < .001).

**Table 2.** Associations Between Predialysis Nephrology Care, KDE, and Incident Access Modality Across the Study Groups

Variable	Referent	Unadjusted model, OR (95% CI)^a^^,^^b^	Adjusted model, aOR (95% CI)^a^^,^^c^
Among Hispanic patients vs White non-Hispanic patients			
Any nephrology care (n = 25 382)	No nephrology care (n = 20 764)	0.67 (0.66-0.69)	0.70 (0.68-0.72)
>6 mo Nephrology care (n = 18 890)	No nephrology care (n = 20 764)	0.64 (0.63-0.66)	0.65 (0.64-0.68)
Nephrology care with predialysis KDE (n = 412)	No KDE (n = 45 733)	0.59 (0.54-0.66)	0.63 (0.56-0.71)
Incident composite AVF or AVG (n = 6625)	CVC only (n = 31 240)	0.76 (0.74-0.78)	0.78 (0.75-0.80)
Incident AVF (n = 5608)	CVC only (n = 31 240)	0.75 (0.72 0.77)	0.77 (0.75-0.80)
Incident AVG (n = 1017)	CVC only (n = 31 240)	0.82 (0.77-0.88)	0.80 (0.74-0.87)
Incident maturing AVF or AVG with CVC (n = 8198)^d^	CVC only (n = 31 240)	1.00 (0.97-1.03)^e^	0.94 (0.91-0.97)
Incident CVC only (n = 31 240)	All other access (n = 14 096)	1.15 (1.12-1.17)	1.17 (1.14-1.20)
Among Black patients vs White non-Hispanic patients			
Any nephrology care (n = 52 001)	No nephrology care (n = 40 886)	0.76 (0.74-0.77)	0.79 (0.77-0.80)
>6 mo Nephrology care (n = 39 112)	No nephrology care	0.73 (0.72-0.75)	0.77 (0.76-0.79)
Nephrology care with predialysis KDE (n = 1180)	No KDE (n = 91 707)	0.85 (0.80-0.91)	0.86 (0.79-0.93)
Incident composite AVF or AVG (n = 16 179)	CVC only (n = 59 475)	0.97 (0.95-0.99)	1.00 (0.98-1.03)^e^
Incident AVF use (n = 11 649)	CVC only (n = 59 475)	0.82 (0.80-0.83)	0.85 (0.83-0.87)
Incident AVG use (n = 4530)	CVC only (n = 59 475)	1.91 (1.84-1.99)	1.87 (1.79-1.96)
Incident maturing AVF or AVG with CVC (n = 17 084)	CVC only (n = 59 475)	1.09 (1.07-1.12)	1.07 (1.05-1.10)
Incident CVC only (n = 59 475)	All other access (n = 33 412)	0.97 (0.96-0.99)	0.97 (0.95-0.98)

Abbreviations: aOR, adjusted odds ratio; AVF, arteriovenous fistula; AVG, arteriovenous graft; CVC, central venous catheter; KDE, kidney replacement therapy–directed kidney disease education; OR, odds ratio.

^a^ *P* < .001 for all analyses except when noted otherwise.

^b^ The ORs for KDE and vascular access modality refer to the prevalence of variable (ie, the predialysis nephrology care, more than 6 months of nephrology care, and predialysis KDE) among the respective groups vs their referent group; the non-Hispanic White individuals served as the referent group for both Black and Hispanic individuals.

^c^ Race and ethnicity models were adjusted for age, sex, diabetes, congestive heart failure, coronary artery disease, body mass index, facility profit status, low-income subsidy indicator, and rurality.

^d^ Maturing accesses refer to an AVF or AVG in situ but not usable on first dialysis.

^e^ Not significant (ie, *P* > .05).

Examining sequential events (Table 3), compared with no predialysis nephrology care, any predialysis nephrology care was associated with a nearly 12-fold (aOR, 11.60; 95% CI, 11.20-12.02) higher odds of incident vascular access, and receiving nephrology care for more than 6 months was associated with 14-fold (aOR, 14.08; 95% CI, 13.50, 14.68) higher odds of incident vascular access. Predialysis KDE service was associated with a 3-fold higher odds of incident vascular access use (aOR, 3.47; 95% CI, 3.23-3.73) vs no predialysis KDE. Significant associations were also found between the individual components of incident AVF and AVG use. Overall, the odds for CVC-only use were 78% lower among those with nephrology care (aOR, 0.22; 95% CI, 0.22-0.23; *P* < .001), with further signal strengthening with longer nephrology care and predialysis KDE services.

**Table 3.** Association Between Sequential Processes of Predialysis Nephrology Care, KDE, and Incident Vascular Access Modality

Outcome	Referent	Unadjusted model, OR (95% CI)^a^	Adjusted model, aOR (95% CI)^a^
Among patients with predialysis nephrology care			
Nephrology care with predialysis KDE (n = 4787)	No KDE (n = 453)	4.08 (3.70-4.49)	3.93 (3.50-4.41)
Incident composite AVF or AVG (n = 66 124)	CVC only (n = 141 571)	11.60 (11.20-12.02)	11.43 (10.97-11.91)
Incident AVF use (n = 55 351)	CVC only (n = 141 571)	14.84 (14.21-15.50)	14.30 (13.60-15.03)
Incident AVG use (n = 10 773)	CVC only (n = 141 571)	5.48 (5.15-5.82)	5.68 (5.29-6.11)
Incident maturing AVF or AVG (n = 53 359)^b^	CVC only (n = 141 571)	2.50 (2.45-2.56)	2.57 (2.51-2.64)
Incident CVC only (n = 141 571)	All other access (n = 119 956)	0.23 (0.22-0.23)	0.22 (0.22-0.23)
Among patients with >6 mo predialysis nephrology care			
Nephrology care with predialysis KDE (n = 4031)	No KDE (n = 453)	4.48 (4.07-4.94)	4.35 (3.87-4.89)
Incident composite AVF or AVG (n = 57 202)	CVC only (n = 100 819)	14.10 (13.60-14.62)	14.08 (13.50-14.68)
Incident AVF use (n = 48 351)	CVC only (n = 100 819)	18.21 (17.43-19.02)	17.77 (16.90-18.68)
Incident AVG (n = 8851)	CVC only (n = 100 819)	6.32 (5.94-6.72)	6.66 (6.19-7.16)
Incident maturing AVF or AVG (n = 42 321)^b^	CVC only (n = 100 819)	2.79 (2.73-2.85)	2.87 (2.79-2.94)
Incident CVC only (n = 100 819)	All other access (n = 99 875)	0.19 (0.19-0.20)	0.19 (0.18-0.19)
Among patients with predialysis KDE			
Incident composite AVF or AVG (n = 2207)	CVC only (n = 2316)	3.58 (3.38-3.80)	3.47 (3.23-3.73)
Incident AVF (n = 1881)	CVC only (n = 2316)	3.70 (3.48-3.94)	3.61 (3.35-3.89)
Incident AVG (n = 326)	CVC only (n = 2316)	3.01 (2.68-3.38)	2.82 (2.44-3.25)
Incident maturing AVF or AVG (n = 1365)^b^	CVC only (n = 2316)	2.21 (2.06-2.36)	2.25 (2.07-2.44)
Incident CVC only (n = 2316)	All other access (n = 3579)	0.35 (0.33-0.37)	0.35 (0.33-0.37)

Abbreviations: aOR, adjusted odds ratio; AVF, arteriovenous fistula; AVG, arteriovenous graft; CVC, central venous catheter; KDE, kidney replacement therapy–directed kidney disease education.

^a^ Refers to the α value for the regression analysis with *P* < .001. Models were adjusted for age, sex, diabetes, congestive heart failure, coronary artery disease, body mass index, facility profit status, low-income subsidy indicator, and rurality.

^b^ Maturing accesses refers to an AVF or AVG in situ but not usable on first dialysis.

Compared with White individuals, total adjusted odds for incident vascular access among Hispanic individuals were 23% lower (aOR, 0.77; 95% CI, 0.75-0.80); however, the aOR for natural direct effects was 0.85 (95% CI, 0.81-0.88), and the aOR for natural indirect effects was 0.91 (95% CI, 0.90-0.92), suggesting that lack of nephrology care mediated one-third (32.59%) of vascular access underuse and 62.00% of maturing vascular access underuse (Table 4), with nearly similar associations with the individual components of incident AVF (32.65%) and AVG (33.80%) underuse. Sensitivity analyses among individuals with more than 6 months of predialysis nephrology care or KDE services revealed further increase in the total and attributable association with vascular access outcomes (Table 4). Similarly, sensitivity analysis using all 733 786 active Medicare recipients at incident hemodialysis showed marginal blunting of total effect but heightened mediation, as hypothesized (eTables 1 and 2 in Supplement 1). Finally, secondary analysis among Black individuals restricted to the disparate outcome of incident AVF showed a lower overall association with incident AVF use (aOR, 0.88; 95% CI, 0.85-0.90) but a higher (53.55%) mediation by the lack of nephrology care in incident AVF underuse (eTable 3 in Supplement 1).

**Table 4.** Attributable Influence of Predialysis Care Disparities on Incident Access Modality Outcomes Among Hispanic vs White Patients^a^

Outcome	Total effect^b^		Natural direct effect (95% CI)^c^		Natural indirect effect (95% CI)^d^		Adjusted percentage mediated^e^	*P* value
	OR (95% CI)	aOR (95% CI)	OR (95% CI)	aOR (95% CI)	OR (95% CI)	aOR (95% CI)		
Primary outcome: composite of incident AVF or AVG								
Any nephrology care	0.76 (0.73-0.78)	0.77 (0.74-0.80)	0.84 (0.81-0.86)	0.85 (0.81-0.88)	0.90 (0.90-0.91)	0.91 (0.90-0.92)	32.59	<.001
>6 mo Nephrology care	0.73 (0.70-0.75)	0.76 (0.72-0.79)	0.84 (0.81-0.87)	0.87 (0.83-0.90)	0.87 (0.86-0.88)	0.87 (0.86-0.89)	44.62	<.001
Nephrology care with predialysis KDE	0.54 (0.47-0.60)	0.59 (0.50-0.67)	0.73 (0.62-0.83)	0.75 (0.63-0.87)	0.74 (0.70-0.79)	0.78 (0.73-0.83)	40.12	<.001
Secondary outcome: incident AVF								
Any nephrology care	0.74 (0.72-0.77)	0.77 (0.74-0.80)	0.83 (0.80-0.86)	0.84 (0.81-0.88)	0.90 (0.89-0.91)	0.91 (0.90-0.92)	32.65	<.001
>6 mo Nephrology care	0.72 (0.69-0.74)	0.75 (0.72-0.79)	0.83 (0.80-0.86)	0.86 (0.83-0.90)	0.86 (0.85-0.87)	0.87 (0.86-0.88)	45.20	<.001
Nephrology care with predialysis KDE	0.51 (0.44-0.58)	0.54 (0.45-0.63)	0.70 (0.59-0.81)	0.71 (0.58-0.84)	0.72 (0.68-0.77)	0.76 (0.71-0.82)	36.64	<.001
Secondary outcome: incident AVG								
Any nephrology care	0.81 (0.76-0.87)	0.80 (0.73-0.87)	0.89 (0.83-0.96)	0.87 (0.79-0.94)	0.91 (0.90-0.92)	0.92 (0.91-0.93)	33.80	<.001
>6 mo Nephrology care	0.77 (0.71-0.83)	0.77 (0.69-0.84)	0.87 (0.80-0.94)	0.87 (0.78-0.95)	0.88 (0.87-0.89)	0.88 (0.87-0.90)	43.22	<.001
Nephrology care with predialysis KDE	0.80 (0.64-0.95)	0.88 (0.67-1.10)	0.88 (0.70-1.05)	0.97 (0.73-1.20)	0.91 (0.86-0.95)	0.92 (0.87-0.97)	69.89	.36
Secondary outcome: incident maturing AVF or AVG with CVC^f^								
Any nephrology care	0.99 (0.96-1.01)	0.93 (0.89-0.96)	1.04 (1.01-1.07)	0.97 (0.94-1.01)	0.95 (0.94-0.95)	0.95 (0.95-0.96)	62.00	<.001
>6 mo Nephrology care	0.98 (0.95-1.01)	0.91 (0.88-0.95)	1.05 (1.02-1.09)	0.98 (0.94-1.03)	0.93 (0.92-0.94)	0.93 (0.92-0.94)	82.53	<.001
Nephrology care with predialysis KDE	1.20 (1.13-1.26)	1.09 (1.01-1.16)	1.24 (1.17-1.31)	1.12 (1.04-1.20)	0.97 (0.95-0.98)	0.97 (0.96-0.99)	−37.70	.04
Secondary outcome: incident CVC only, vs all else combined								
Any nephrology care	1.04 (1.00-1.07)	1.08 (1.03-1.12)	0.92 (0.89-0.95)	0.96 (0.92-1.00)	1.13 (1.12-1.14)	1.12 (1.11-1.13)	151.46	<.001
>6 mo Nephrology care	1.04 (1.00-1.08)	1.08 (1.03-1.14)	0.89 (0.86-0.92)	0.93 (0.89-0.97)	1.16 (1.15-1.18)	1.16 (1.15-1.18)	184.49	.001
Nephrology care with predialysis KDE	0.83 (0.79-0.87)	0.90 (0.84-0.96)	0.81 (0.77-0.85)	0.88 (0.82-0.94)	1.03 (1.03-1.03)	1.02 (1.02-1.03)	−21.39	.005

Abbreviations: aOR, adjusted odds ratio; AVF, arteriovenous fistula; AVG, arteriovenous graft; CVC, central venous catheter; KDE, kidney replacement therapy–directed kidney disease education.

^a^ The procedure estimates mediation effects from a set of relationships between an outcome variable (vascular access modality use), a treatment variable (race/ethnicity), a mediator variable (nephrology care, more than 6 months nephrology care, KDE), and a set of background covariates (age, sex, diabetes, congestive heart failure, coronary artery disease, body mass index, facility profit status, low-income subsidy indicator, rurality). For the outcomes of AVF, AVG, composite of AVF and AVG and maturing AVF and AVG we used CVC-only use as the reference. For the CVC-only population, all other mature and maturing accesses were used as reference.

^b^ The total effect equals the natural direct effect multiplied by the natural indirect effect.

^c^ The natural direct effect refers to the direct effect of race and ethnicity on vascular access in absence of the mediator.

^d^ The natural indirect effect refers to the effect of race and ethnicity on vascular access utilization through the mediator.

^e^ The adjusted mediation percentage is the percentage of the adjusted total effect that is attributable to the natural indirect effect.

^f^ Maturing accesses refer to an AVF or AVG in situ but not usable on first dialysis.

In secondary analysis among those initiating hemodialysis with CVC with or without maturing AVF or AVG (Table 5 and eTable 4 in Supplement 1), compared with White individuals, Hispanic individuals with CVC and a maturing AVF or AVG had 38% (aOR, 1.38; 95% CI, 1.23-1.53) higher odds and those with CVC only had 30% (aOR, 1.30; 95% CI, 1.25-1.35) higher odds of conversion to a functional AVF or AVG within the first year of dialysis. Examining these through the mediation model revealed that predialysis nephrology care negatively mediated conversion to mature vascular access.

**Table 5.** Attributable Association of Disparities in Predialysis Nephrology Care With CVC to Arteriovenous Vascular Access Conversions During the First Year Receiving Dialysis Among Hispanic vs White Individuals^a^

Variable	Total effect^b^		Natural direct effect (95% CI)^c^		Natural indirect effect (95% CI)^d^		Adjusted percentage mediated^e^	*P* value
	OR (95% CI)	aOR (95% CI)	OR (95% CI)	aOR (95% CI)	OR (95% CI)	aOR (95% CI)		
Switch to AVF or AVG among those with incident CVC only^f^								
Any nephrology care	1.32 (1.27-1.36)	1.30 (1.25-1.35)	1.35 (1.30-1.39)	1.33 (1.27-1.38)	0.98 (0.97-0.98)	0.98 (0.97-0.98)	−8.95	<.001
>6 mo Nephrology care	1.28 (1.23-1.32)	1.28 (1.22-1.34)	1.32 (1.27-1.37)	1.32 (1.26-1.38)	0.97 (0.96-0.98)	0.97 (0.96-0.98)	−14.72	<.001
Nephrology care with predialysis KDE	1.32 (1.25-1.38)	1.30 (1.22-1.39)	1.34 (1.27-1.41)	1.33 (1.24-1.41)	0.99 (0.97-1.00)	0.98 (0.97-1.00)	−7.07	.02
Switch to AVF or AVG among those with incident maturing AVF or AVG^f^^,^^g^								
Any nephrology care	1.33 (1.22-1.44)	1.38 (1.23-1.53)	1.36 (1.25-1.48)	1.41 (1.26-1.57)	0.97 (0.96-0.99)	0.98 (0.96-0.99)	−8.86	.002
>6 mo Nephrology care	1.35 (1.22-1.47)	1.41 (1.24-1.58)	1.40 (1.26-1.54)	1.47 (1.28-1.65)	0.96 (0.95-0.98)	0.97 (0.95-0.98)	−12.29	.001
Nephrology care with predialysis KDE	1.24 (1.04-1.44)	1.22 (0.96-1.48)	1.29 (1.05-1.53)	1.28 (0.95-1.61)	0.96 (0.89-1.03)	0.95 (0.85-1.05)	−28.77	.82

Abbreviations: aOR, adjusted odds ratio; AVF, arteriovenous fistula; AVG, arteriovenous graft; CVC, central venous catheter; KDE, kidney replacement therapy–directed kidney disease education.

^a^ The procedure estimates mediation effects from a set of relationships between an outcome variable (switching to AVF or AVG from CVC), a treatment variable (race and ethnicity), a mediator variable (nephrology care, more than 6 months nephrology care, or KDE), and a set of background covariates (age, sex, diabetes, congestive heart failure, coronary artery disease, body mass index, facility profit status, low-income subsidy indicator, and rurality).

^b^ The total effect equals the natural direct effect multiplied by the natural indirect effect.

^c^ The natural direct effect refers to the direct effect of race or ethnicity on vascular access in the absence of the mediator.

^d^ The natural indirect effect refers to the effect of race or ethnicity on switching to AVF/AVG through the mediator.

^e^ The adjusted mediation percentage is the percent of the adjusted total effect that is attributable to the natural indirect effect.

^f^ Switch to AVF/AVG refers to those with incident CVC with or without maturing arteriovenous access, who are able to use AVF or AVG during the first year on dialysis.

^g^ Maturing accesses refer to an AVF or AVG in situ but not usable on first dialysis.

## Discussion

To our knowledge, this cohort study is the first of its kind among Medicare recipients undergoing incident hemodialysis. We found that Hispanic patients have 23% lower rates of incident vascular access compared with White individuals. Approximately one-third of this disparity is attributable to their lack of access to predialysis nephrology care, with further increasing association when analyzed among all Medicare recipients and those with longer duration of predialysis nephrology care or KDE services. Finally, we found that with the availability of universal nephrology care during the first year receiving dialysis, Hispanic individuals converted to mature vascular access at significantly higher rates. These findings have important public health implications.

The CMS National Quality Forum has recognized incident vascular access rates as a core quality metric for hemodialysis patients owing to its association with longitudinal patient outcomes.^3,4^ Although it is assessed during dialysis, incident vascular access is dependent on predialysis care.^8^ By analyzing the USRDS, Zarkowsky et al^10^ found that Hispanic individuals have among the lowest vascular access outcomes across all racial and ethnic groups in the US, with 32% lower rates of incident AVF than White individuals. Purnell et al^12^ showed that although there have been generalized improvements in predialysis nephrology care over the last 2 decades, disparities have continued unabated for marginalized populations; Hispanic individuals have 39% lower odds of receiving predialysis nephrology care than White individuals. Recently, we showed similar trends for predialysis KDE provision, with 22% lower odds of KDE among Hispanic than White individuals.^17^ Epidemiological studies have long demonstrated associations between these care types and vascular access outcomes; patients with predialysis nephrology care have 11-fold higher odds,^16^ and predialysis KDE services nearly 2-fold higher odds, independently of and in addition to nephrology care, of incident vascular access outcomes.^17^ These cross-sectional associations indicate, but do not explain or quantify, the magnitude of attributable association of these sequential disparities.

Mediation analyses can dissect sequential multifactorial associations to quantify the attributable association of mediators with outcomes. This is especially relevant when studying health disparities, where randomized studies are not feasible.^19^ Using robust methods to control well-known biological and social determinants associated with vascular access outcomes,^10,14^ for the first time, we quantify the attributable association of predialysis nephrology care with incident vascular access underuse among Hispanic individuals. Furthermore, our extensive sensitivity analyses modulating key covariates and mediators ensure the correct interpretation of our findings. For example, given the association between predialysis health care insurance and incident vascular access outcomes, we restricted our primary analysis to individuals with at least 6 months of predialysis Medicare coverage and performed a sensitivity analysis that included all active Medicare recipients. As expected, broadening the population further increased the attributable influence of predialysis care disparities on incident vascular access outcomes. Second, our sensitivity analyses show larger attributable associations with increasing mediator strength and quality, providing additional confirmation for our interpretations and explanation for prior epidemiological analyses. A recent Dialysis Outcomes and Practice Patterns Study–III analysis showed a substantial association between predialysis nephrology care duration and incident vascular access outcomes; 77% of patients with less than 1 month of nephrology care initiated dialysis with CVC, compared with only 36% receiving 4 or more months.^20^ Third, our secondary analysis evaluating disparate AVF outcomes among Black individuals further confirms the directionality and importance of predialysis nephrology care. Finally, our demonstration that compared with White individuals, Hispanic individuals with incident CVC convert to AVF or AVG at significantly higher rates during their first year on dialysis, when everyone is under nephrology care, and more importantly, that predialysis care is negatively associated with these conversions, suggests a potential care-naive population and the importance of predialysis nephrology care. Together, these analyses show that a substantial proportion of incident vascular access disparities among Hispanic individuals is not related to biological limitations but can be better understood in the context of their lack of access to predialysis care.

Our analysis also clarifies the association of predialysis KDE with vascular access outcomes. Qualitative studies have shown that some patients undergoing hemodialysis may prefer CVC over arteriovenous access, potentially for their identity or lifestyle.^21^ Considering the limited survival gains with arteriovenous access among older, frail patients and those with high comorbidity burden, the 2019 Kidney Disease Outcomes Quality Initiative update proposed a patient-centered, “right access, in right patient, at right time, for right reasons” approach to finalize individuals’ vascular access.^5^ A few recent studies have indicated conflicting results when using such a patient-centered approach. Analyzing USRDS, Allon et al^22^ recently reported declining trends for incident AVF between 2015 and 2020. They posited that, in addition to high home dialysis and conservative care use, patient-centered vascular access practices may have caused patients undergoing hemodialysis to use CVC in higher proportions. Considering the limitations of coding practices in capturing patient-centered practices, such as KDE, we acknowledge that our analysis cannot definitively capture the association of KDE with vascular access outcomes. Nonetheless, our findings clearly establish the positive directionality and significant proportional association of predialysis KDE with vascular access outcomes. Our findings are also aligned with more granular institution-level data; Hanko et al^23^ showed that implementing KRT-directed education substantially increases the odds of all incident kidney failure outcomes (ie, home dialysis, transplantations, and vascular access rates). However, these benefits are blunted among those unable to achieve KRT-related decisions after education, with vascular access rates in these groups reported to be 50% vs 79%, respectively.

Taken together, our results highlight an essential distinction: early indications that incident vascular access had substantial associations and a potential causal relationship with on-hemodialysis survival were instrumental in regulatory agencies establishing aspirational incident vascular access rates as a predialysis care quality metric.^3,4^ Recognizing inherent bias in these assumptions concerning the limitations imposed by individuals’ comorbidity status, recent guidelines have revised their recommendations to advocate shared decision-making to determine individuals’ vascular access.^5^ Unfortunately, these broad categories have a mutually contradictory influence on survival. Although aiming for high incident vascular access targets may harm individuals who are old, frail, or have significant comorbidities, the guidelines still emphasize identifying concerns that reflect quality and could impact survival. Predialysis nephrology care and its duration have a substantial and inverse association with kidney failure mortality.^16^ Hispanic individuals have lower mortality while undergoing dialysis than White individuals, likely because they are younger and have lower comorbidity burden, exactly the factors implicated in their higher odds of vascular access intervention success seen in prior studies^24,25^ and our secondary analysis. Moreover, a recent analysis^15^ found that predialysis care disparities have a similar attributable association with home dialysis utilization, an equally important measure defining optimal kidney replacement therapy initiation in CMS categorization^26^ and a major focus for the US health care system.^27^ Pending studies that carefully examine the impact of these incident kidney failure disparities on post–kidney failure survival in this otherwise younger and healthier cohort, system-based interventions are needed to identify and address the root causes of these care disparities, which can allow us leverage the clinical and health care cost benefits of improving predialysis care on optimal KRT initiation.

### Limitations

Although we found that a sizable portion of incident vascular access disparity among Hispanic individuals can be attributed to the upstream system-level factor of access to care, a substantial residual disparity remains. These residual associations are likely related to various practitioner-related and patient-related factors, which are difficult to dissect in large database analyses. Prior studies^18,28,29^ have shown that patient-level factors, such as social determinants of health, trust, and response patterns among marginalized populations, and practitioner limitations and bias are associated with the provision of quality care and health care outcomes. Examining their attributable association will likely require targeted, potentially qualitative evaluations. In addition, the significant interactions between race, ethnicity, mediator, and outcomes in our findings suggest that the mediators, predialysis nephrology care and KDE, differentially impact vascular access outcomes among marginalized populations, potentially pointing toward the complexity of practitioner and patient behavior, including language and cultural discordance, which may drive some of these processes.^30,31^ Second, to ensure that we evaluate the impact of disparity among those with presumed equal access and avoid bias related to the time required for patient care, education, and medical procedures on incident vascular access, we restricted our analysis to those with Medicare insurance coverage. Although it is mechanistically desirable, avoiding the heterogeneous populations with private or Medicaid insurance or younger age may have blunted the attributable associations seen in our analysis. Moreover, the mediation stipulations established by Baron et al^18^ limited us from using the mediation model on the primary composite of AVF and AVG for Black individuals, and thus, limit firm conclusions. Nonetheless, the congruity of our secondary analysis establishing similarly significant attributable associations with AVF use disparity among Black compared with White individuals strengthens our primary conclusions and argues for additional multimediation model studies to better dissect the heterogeneous association of disparity among the Black population. Third, our study examined multiple outcomes, and such multiple comparisons may result in a high risk of type I error. We applied an adjusted significance threshold of *P* < .01 to account for these multiple comparisons.

## Conclusions

In this retrospective cohort study of patients undergoing incident hemodialysis, we found that despite being younger and having less comorbidity burden, Hispanic individuals had lower incident vascular access rates than White patients. We further demonstrated the temporality and directionality of structural inequities, showing that the roots of incident vascular access disparities were long-standing and complex. Together, our results suggest that access to care is a major barrier to health care equity, arguing for greater attention from payers and nonnephrology practitioners toward processes that facilitate earlier recognition and nephrology referrals for patients with advanced chronic kidney disease. Ultimately, these results underscore the need for more mechanistic, hypothesis-driven mediation studies to gain a deeper understanding of the prominent factors associated with kidney failure outcomes.

## Notes

Supplement 1. **eFigure 1.** Conceptual Model for Causal Mediation Analysis Derived From the Model for Advanced CKD Care in the US **eFigure 2.** Patient Selection Diagram **eTable 1.** Demographics and Clinical Characteristics of All Patients With Active Medicare Coverage at Dialysis Initiation Across Ethnicity/Race Groups **eTable 2.** Attributable Influence of Disparities in Pre-dialysis Care on Incident Vascular Access Outcomes Among All Hispanic Compared to White Active Medicare Recipients at Hemodialysis Initiation **eTable 3.** Attributable Influence of Disparities in Pre-dialysis Care on Arteriovenous Fistula Among Black Compared to White Patients With at Least 6 Months of Pre-dialysis Medicare Coverage **eTable 4.** Attributable Influence of Disparities in Pre-dialysis Nephrology Care on Central Venous Catheter to Arteriovenous Vascular Access Conversions During the First Year on Dialysis Among Hispanic Compared to White Active Medicare Recipients at Hemodialysis Initiation

Supplement 2.
Data Sharing Statement
