# Supplementary material for: Predialysis Nephrology Care Disparities and Incident Vascular Access Among Hispanic Individuals
Source: JAMA Netw Open. 2025 Sep 5;8(9):e2530972. doi: 10.1001/jamanetworkopen.2025.30972 (PMC12413648; doi:10.1001/jamanetworkopen.2025.30972)
Supplement: Supplement 1. — eFigure 1. Conceptual Model for Causal Mediation Analysis Derived From the Model for Advanced CKD Care in the US eFigure 2. Patient Selection Diagram eTable 1. Demographics and Clinical Characteristics of All Patients With Active Medicare Coverage at Dialysis Initiation Across Ethnicity/Race Groups eTable 2. Attributable Influence of Disparities in Pre-dialysis Care on Incident Vascular Access Outcomes Among All Hispanic Compared to White Active Medicare Recipients at Hemodialysis Initiation eTable 3. Attributable Influence of Disparities in Pre-dialysis Care on Arteriovenous Fistula Among Black Compared to White Patients With at Least 6 Months of Pre-dialysis Medicare Coverage eTable 4. Attributable Influence of Disparities in Pre-dialysis Nephrology Care on Central Venous Catheter to Arteriovenous Vascular Access Conversions During the First Year on Dialysis Among Hispanic Compared to White Active Medicare Recipients at Hemodialysis Initiation [file jamanetwopen-e2530972-s001.pdf]

Supplemental Online Content

Pramod S, Scheiffele G, Huang W, et al. Predialysis nephrology care disparities and incident vascular access among Hispanic individuals. *JAMA Netw Open*. 2025;8(9):e2530972.
doi:10.1001/jamanetworkopen.2025.30972

eFigure 1. Conceptual Model for Causal Mediation Analysis Derived From the Model for Advanced CKD Care in the US

eFigure 2. Patient Selection Diagram

eTable 1. Demographics and Clinical Characteristics of All Patients With Active Medicare Coverage at Dialysis Initiation Across Ethnicity/Race Groups

eTable 2. Attributable Influence of Disparities in Pre-dialysis Care on Incident Vascular Access Outcomes Among All Hispanic Compared to White Active Medicare Recipients at Hemodialysis Initiation

eTable 3. Attributable Influence of Disparities in Pre-dialysis Care on Arteriovenous Fistula Among Black Compared to White Patients With at Least 6 Months of Pre-dialysis Medicare Coverage

eTable 4. Attributable Influence of Disparities in Pre-dialysis Nephrology Care on Central Venous Catheter to Arteriovenous Vascular Access Conversions During the First Year on Dialysis Among Hispanic Compared to White Active Medicare Recipients at Hemodialysis Initiation

This supplemental material has been provided by the authors to give readers additional information about their work.

© 2025 Pramod S et al. *JAMA Network Open*.

eFigure 1: Conceptual Model for Causal Mediation Analysis Derived From the Model for Advanced CKD Care in the US

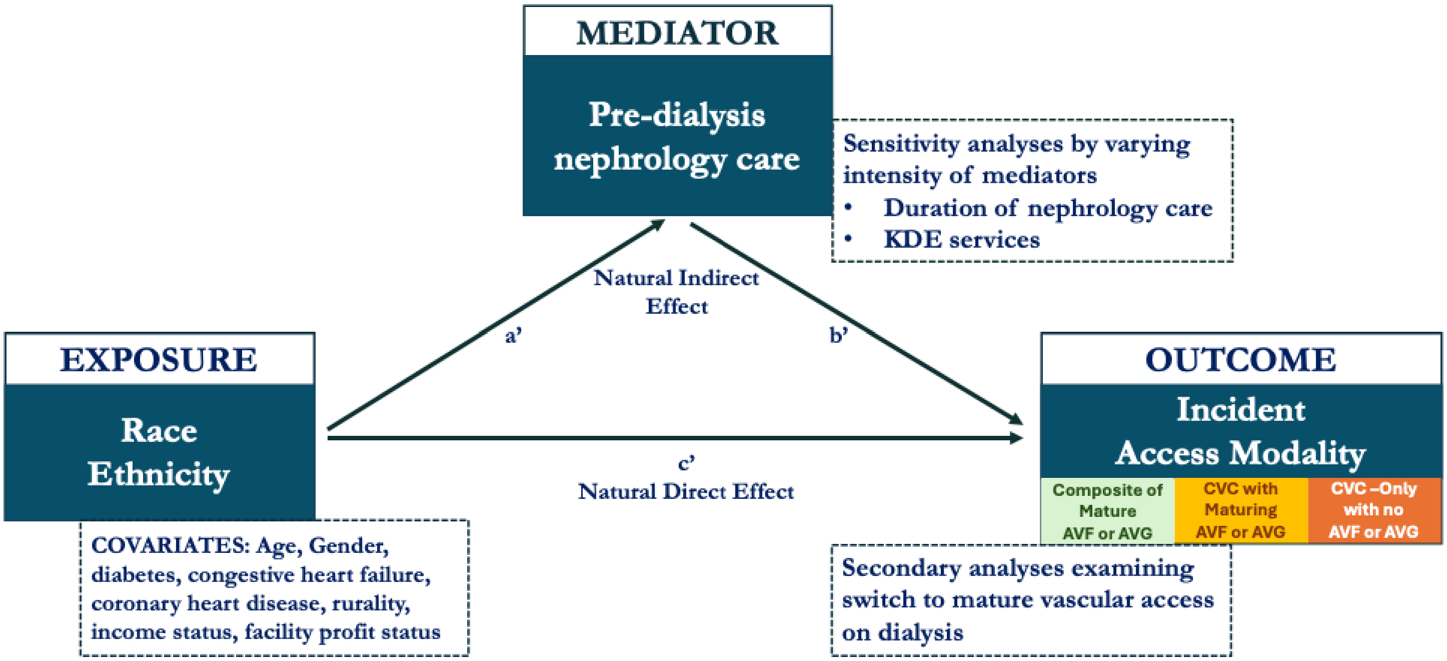

eFigure 1 notes: BMI: body mass index; KDE: KRT-directed education. The natural indirect effect refers to the effect of disparity on vascular access outcomes that is mediated by affecting the occurrence of pre-dialysis nephrology care. The natural direct effect is the residual or direct effect of disparities on vascular access outcomes that is not mediated by pre-dialysis nephrology care.

© 2025 Pramod S et al. *JAMA Network Open*.

eFigure 2: Patient Selection Diagram:

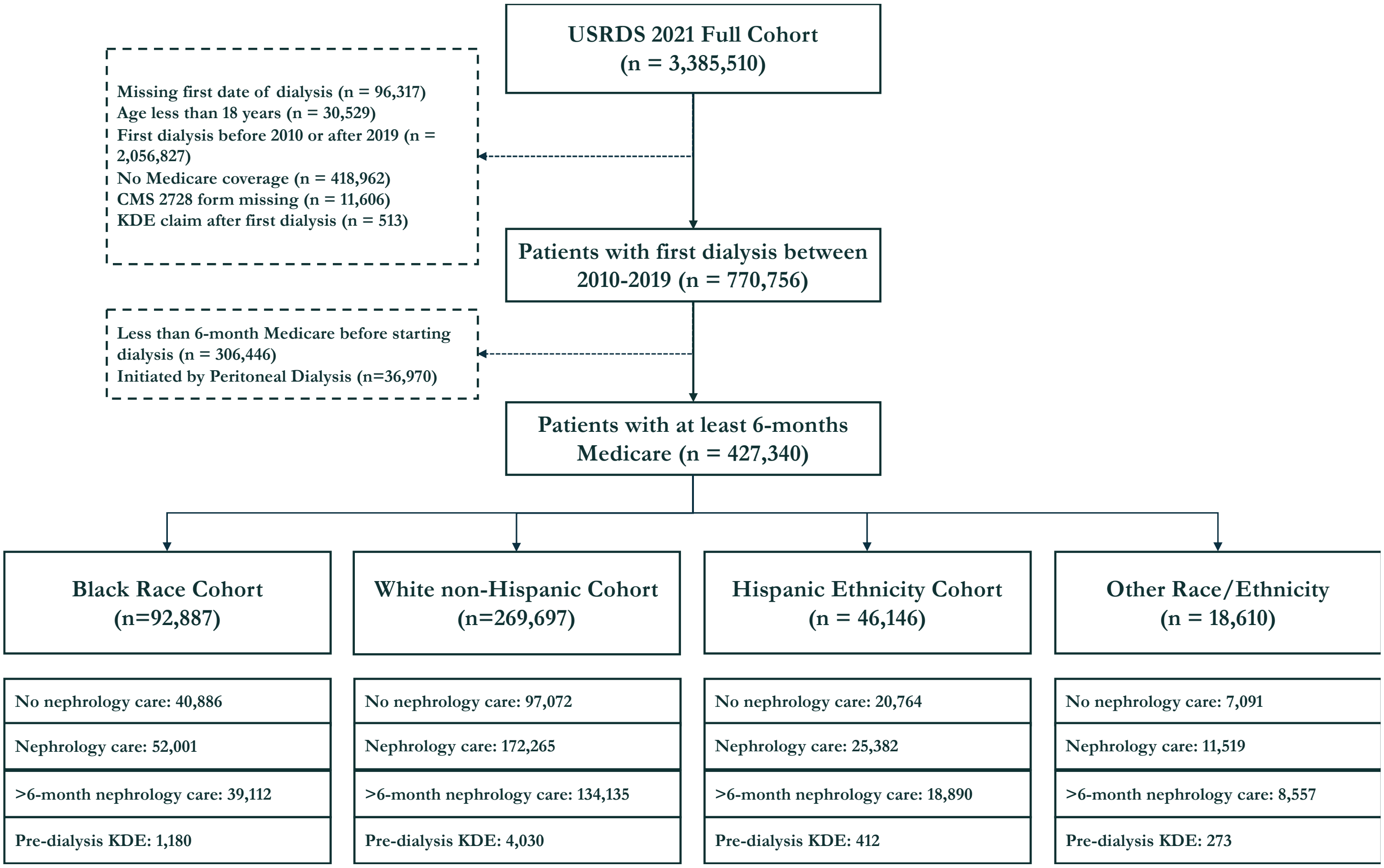

eFigure 2 notes: USRDS: United States Renal Database System; CMS: Center for Medicare and Medicaid Services; KDE: KRT-directed education

© 2025 Pramod S et al. *JAMA Network Open*.

eTable 1: Demographics and Clinical Characteristics of All Patients With Active Medicare Soverage at Dialysis Initiation Across Ethnicity/Race Groups

Patient Characteristics	White non-Hispanic (n=405,301)	Hispanic ethnicity (n = 83,302)	Black Race (n=167,262)
Age group			
18-44 years	8,309 (2.1%)	2,847 (3.4%)	8,149 (4.9%)
45-64 years	70,266 (17.3%)	20,196 (24.2%)	46,855 (28.0%)
>65 years	326,726 (80.6%)	60,259 (72.3%)	112,258 (67.1%)
Sex			
Female	164,867 (40.7%)	34,674 (41.7%)	83,852 (50.3%)
Male	240,434 (59.3%)	48,628 (58.3%)	83,140 (49.7%)
Employment status			
Employed	15,939 (3.9%)	2,320 (2.8%)	5,690 (3.4%)
Retired	338,599 (83.5%)	61,694 (74.1%)	125,854 (75.2%)
Unemployed/ other	50,763 (12.5%)	19,288 (23.2%)	35,718 (21.4%)
Primary Disease			
Diabetes	181,410 (44.8%)	55,192 (66.3%)	79,712 (47.7%)
Hypertension	122,920 (30.3%)	17,928 (21.5%)	64,652 (38.7%)
Glom. Nephritis	22,250 (5.5%)	2,825 (3.4%)	6,354 (3.8%)
Cystic Kidney Disease	5,902 (1.5%)	520 (0.6%)	1,197 (0.7%)
Other Urologic Diseases	7,763 (1.9%)	732 (0.9%)	1,068 (0.6%)
Other Causes	55,007 (13.6%)	4,988 (6.0%)	11,371 (6.8%)
Unknown cause	10,133 (2.5%)	1,064 (1.3%)	2,908 (1.7%)
Comorbidities			
Diabetes	247,951 (61.2%)	65,676 (78.8%)	112,478 (67.2%)
Congestive Heart Failure	154,778 (38.2%)	26,726 (32.1%)	58,095 (34.7%)
Hypertension	348,725 (86.0%)	74,700 (89.7%)	152,082 (90.9%)
COPD	62,910 (15.5%)	5,322 (6.4%)	16,445 (9.8%)
Peripheral Vascular Disease	59,425 (14.7%)	13,088 (15.7%)	18,017 (10.8%)
Coronary Artery Disease	91,591 (22.6%)	16,168 (19.4%)	23,187 (13.9%)
Cerebrovascular Disease	41,668 (10.3%)	8,023 (9.6%)	21,611 (12.9%)
Albumin (mean SD)	3.16 (1.44)	3.13 (2.78)	3.20 (3.15)
BMI >30	163,976 (40.5%)	29,046 (34.9%)	68,088 (40.7%)
Needs assistance	70,246 (17.3%)	16,042 (19.3%)	28,920 (17.3%)
Inability to ambulate	38,512 (9.5%)	8,140 (9.8%)	16,380 (9.8%)
Facility for-profit status			
For profit Facility	279,773 (69.0%)	59,296 (71.2%)	114,277 (68.3%)
Non-profit Facility	41,290 (10.2%)	5,031 (6.0%)	14,306 (8.6%)
Low-income subsidy recipients	216,310 (53.4%)	50,432 (60.5%)	95,126 (56.9%)
Rurality			
Metropolitan	281,079 (69.4%)	63,395 (76.1%)	135,712 (81.1%)
Micropolitan	48,249 (11.9%)	4,633 (5.6%)	12,305 (7.4%)
Rural	39,015 (9.6%)	2,419 (2.9%)	8,968 (5.4%)
Missing	36,954 (9.1%)	12,855 (15.4%)	10,277 (6.1%)
Pre-dialysis Nephrology Care [a]			
No nephrology care	137,314 (33.9%)	34,669 (41.6%)	66,576 (39.8%)
Any nephrology care	267,987 (66.1%)	48,633 (58.4%)	100,686(60.2%)
More than 6 months care	210,570 (52.0%)	36,889 (44.3%)	77,512 (46.3%)
Pre-dialysis KDE [a]	5,524 (1.4%)	698 (1.0%)	2,069 (1.2%)
Incident Vascular Access Modality [a]			
Incident Composite AVF/AVG	84,200 (20.8%)	14,640 (17.6%)	35,108 (21.0%)
Incident AVF	73,112 (18.1%)	12,706 (15.3%)	26,246 (15.7%)
Incident AVG	11,088 (2.7%)	1,934 (2.3%)	8,862 (5.3%)
Incident Maturing AVF/AVG	251,395 (62.0%)	53,444 (64.2%)	100,397 (60.0%)
Incident CVC-only	68,795 (17.0%)	15,052 (18.1%)	31,408 (18.8%)

eTable 1 notes: KDE: kidney replacement therapy-directed education; AVF: arteriovenous fistula; AVG: arteriovenous graft; CVC: central venous catheter; numbers in the individual column represent n (%), except specifically mentioned; [a] all except Incident Access Modality parameters within the cohort were found to be statistically significantly different with p<0.001 in comparison to the non-Hispanic White individuals. The comparison for the Incident Access Modality is available in Table 2.

© 2025 Pramod S et al. *JAMA Network Open*.

eTable 2: Attributable Influence of Disparities in Pre-dialysis Care on Incident Vascular Access Outcomes Among All Hispanic Compared to White Active Medicare Recipients at Hemodialysis Initiation.

	Total Effect		Natural Direct Effect (95%CI)		Natural Indirect effect (95% CI)		Adjusted Percentage Mediated	p value*
	OR (95%CI)	aOR (95%CI)	OR (95%CI)	aOR (95%CI)	OR (95%CI)	aOR (95%CI)		
**Composite of AVF and AVG Outcome**								
Any Nephrology Care	0.82 (0.80, 0.84)	0.81 (0.79, 0.84)	0.89 (0.87, 0.91)	0.88 (0.85, 0.90)	0.92 (0.92, 0.93)	0.93 (0.92, 0.93)	35.2	<0.001
More than 6-months Nephrology Care	0.79 (0.77, 0.81)	0.79 (0.76, 0.81)	0.88 (0.86, 0.90)	0.88 (0.85, 0.91)	0.90 (0.89, 0.90)	0.90 (0.89, 0.90)	43.17	<0.001
Nephrology care with pre-dialysis KDE	0.81 (0.79, 0.83)	0.81 (0.79, 0.83)	0.82 (0.81, 0.84)	0.82 (0.80, 0.84)	0.98 (0.98, 0.99)	0.99 (0.98, 1.00)	5.59	<0.001
**Arteriovenous Fistula Outcome**								
Any Nephrology Care	0.82 (0.80, 0.84)	0.82 (0.79, 0.84)	0.89 (0.87, 0.91)	0.89 (0.86, 0.91)	0.92 (0.92, 0.93)	0.92 (0.92, 0.93)	37.23	<0.001
More than 6-months Nephrology Care	0.79 (0.77, 0.81)	0.79 (0.77, 0.82)	0.88 (0.86, 0.91)	0.89 (0.86, 0.92)	0.89 (0.89, 0.90)	0.89 (0.89, 0.90)	45.70	<0.001
Nephrology care with pre-dialysis KDE	0.81 (0.79, 0.83)	0.82 (0.79, 0.84)	0.82 (0.80, 0.84)	0.83 (0.80, 0.85)	0.98 (0.98, 0.99)	0.99 (0.98, 0.99)	5.75	<0.001
**Arteriovenous Graft Outcome**								
Any Nephrology Care	0.82 (0.77, 0.86)	0.77 (0.72, 0.83)	0.89 (0.83, 0.93)	0.83 (0.78, 0.89)	0.93 (0.92, 0.94)	0.93 (0.92, 0.94)	26.46	<0.001
More than 6-months Nephrology Care	0.78 (0.74, 0.83)	0.74 (0.68, 0.79)	0.87 (0.82, 0.92)	0.82 (0.76, 0.88)	0.87 (0.82, 0.92)	0.90 (0.89, 0.91)	32.02	<0.001
Nephrology care with pre-dialysis KDE	0.82 (0.78, 0.86)	0.78 (0.73, 0.83)	0.83 (0.79, 0.87)	0.79 (0.74, 0.83)	0.99 (0.99, 1.00)	0.99 (0.99, 1.00)	2.11	<0.001
**Maturing accesses with CVC vs CVC only**								
Any Nephrology Care	0.98 (0.96, 1.01)	1.04 (1.00, 1.08)	0.88 (0.86, 0.90)	0.93 (0.90, 0.96)	1.12 (1.11, 1.13)	1.12 (1.11, 1.13)	264.9	0.02
More than 6-months Nephrology Care	0.98 (0.95, 1.01)	1.05 (1.01, 1.09)	0.86 (0.83, 0.88)	0.91 (0.88, 0.94)	1.15 (1.14, 1.16)	1.15 (1.14, 1.16)	293.7	0.02
Nephrology care with pre-dialysis KDE	1.09 (1.07, 1.11)	1.13 (1.11, 1.15)	1.09 (1.07, 1.10)	1.13 (1.11, 1.15)	1.00 (1.00, 1.00)	1.00 (1.00, 1.00)	2.72	<0.001
**CVC-only Outcome**								
Any Nephrology Care	1.02 (0.99, 1.04)	0.94 (0.91, 0.96)	1.06 (1.04, 1.09)	0.98 (0.95, 1.01)	0.96 (0.95, 0.96)	0.96 (0.95, 0.96)	69.49	<0.001
More than 6-months Nephrology Care	1.00 (0.98, 1.03)	0.91 (0.88, 0.94)	1.07 (1.04, 1.09)	0.98 (0.94, 1.01)	0.94 (0.94, 0.94)	0.94 (0.93, 0.94)	71.37	<0.001
Nephrology care with pre-dialysis KDE	1.03 (1.01, 1.05)	0.95 (0.93, 0.98)	1.03 (1.01, 1.05)	0.96 (0.93, 0.98)	1.00 (0.99, 1.00)	1.00 (0.99, 1.00)	7.91	0.01

eTable2 notes: KDE: KRT-directed kidney disease education; AVF: Arteriovenous Fistula; AVG: Arteriovenous Graft; CVC: Central Venous Catheter; Maturing accesses refer to an AVF or AVG in situ but not usable on first dialysis; aOR: adjusted odds ratio; 95%CI: 95% confidence interval.

The procedure estimates mediation effects from a set of relationships between an outcome variable (Vascular access modality use), a treatment variable (Race/Ethnicity), a mediator variable (Nephrology care, more than 6 months nephrology care, KDE) and a set of background covariates (age, sex, diabetes, congestive heart failure, coronary artery disease, body mass index, facility profit status, low-income subsidy indicator, rurality). For the outcomes of AVF, AVG, composite of AVF and AVG and maturing AVF and AVG we used CVC-only use as the reference. For the CVC-only population, all other mature and maturing accesses were used as reference.

The Natural Direct Effect refers to the direct effect of Race/Ethnicity on vascular access in absence of the Mediator. The Natural Indirect Effect refers to the effect of Race/Ethnicity on Vascular access utilization through the mediator. The Total Effect = (Natural Direct Effect) x (Natural Indirect Effect). The adjusted mediation percentage is the percent of the adjusted Total Effect which is attributable to the Natural Indirect Effect.

© 2025 Pramod S et al. *JAMA Network Open*.

eTable 3: Attributable Influence of Disparities in Pre-dialysis Care on Arteriovenous Fistula Among Black Compared to White Patients With at Least 6 Months of Pre-dialysis Medicare Coverage

	Total Effect		Natural Direct Effect (95%CI)		Natural Indirect effect (95% CI)		Adjusted Percentage Mediated	p value*
	OR (95%CI)	aOR (95%CI)	OR (95%CI)	aOR (95%CI)	OR (95%CI)	aOR (95%CI)		
**Arteriovenous Fistula Outcome among Black Individuals**								
Any Nephrology Care	0.84 (0.82, 0.87)	0.88 (0.85, 0.90)	0.92 (0.89, 0.94)	0.94 (0.92, 0.97)	0.92 (0.92, 0.93)	0.93 (0.92, 0.94)	53.55	<0.0001
More than 6-months Nephrology Care	0.82 (0.79, 0.84)	0.85 (0.82, 0.88)	0.92 (0.89, 0.94)	0.94 (0.90, 0.97)	0.89 (0.89, 0.90)	0.91 (0.90, 0.92)	56.88	<0.0001
Nephrology care with pre-dialysis KDE	0.67 (0.61, 0.74)	0.69 (0.61, 0.77)	0.77 (0.69, 0.84)	0.76 (0.67, 0.85)	0.88 (0.85, 0.91)	0.90 (0.87, 0.94)	23.93	<0.0001

eTable 3 notes: KDE: KRT-directed kidney disease education; AVF: Arteriovenous Fistula; AVG: Arteriovenous Graft; CVC: Central Venous Catheter; Maturing accesses refer to an AVF or AVG in situ but not usable on first dialysis; aOR: adjusted odds ratio; 95%CI: 95% confidence interval.

The procedure estimates mediation effects from a set of relationships between an outcome variable (Vascular access modality use), a treatment variable (Race/Ethnicity), a mediator variable (Nephrology care, more than 6 months nephrology care, KDE) and a set of background covariates (age, sex, diabetes, congestive heart failure, coronary artery disease, body mass index, facility profit status, low-income subsidy indicator, rurality). For the outcomes of AVF, AVG, composite of AVF and AVG and maturing AVF and AVG we used CVC-only use as the reference. For the CVC-only population, all other mature and maturing accesses were used as reference.

The Natural Direct Effect refers to the direct effect of Race/Ethnicity on vascular access in absence of the Mediator. The Natural Indirect Effect refers to the effect of Race/Ethnicity on Vascular access utilization through the mediator. The Total Effect = (Natural Direct Effect) x (Natural Indirect Effect). The adjusted mediation percentage is the percent of the adjusted Total Effect which is attributable to the Natural Indirect Effect.

© 2025 Pramod S et al. *JAMA Network Open*.

eTable 4: Attributable Influence of Disparities in Pre-dialysis Nephrology Care on Central Venous Catheter to Arteriovenous Vascular Access Conversions During the First Year on Dialysis Among Hispanic Compared to White Active Medicare Recipients at Hemodialysis Initiation.

	Total Effect		Natural Direct Effect (95%CI)		Natural Indirect effect (95% CI)		Adjusted Percentage Mediated	p value*
	OR (95%CI)	aOR (95%CI)	OR (95%CI)	aOR (95%CI)	OR (95%CI)	aOR (95%CI)		
Switch to AVF/AVG among Hispanic Individuals with Incident Sole CVC								
Any Nephrology Care	1.05 (0.99, 1.10)	1.08 (1.01, 1.16)	1.07 (1.01, 1.13)	1.10 (1.02, 1.18)	0.98 (0.97, 0.99)	0.98 (0.98, 0.99)	-20.17	0.02
More than 6-months Nephrology Care	1.01 (0.95, 1.07)	1.08 (1.00, 1.16)	1.04 (0.97, 1.10)	1.11 (1.02, 1.20)	0.97 (0.97, 0.98)	0.98 (0.97, 0.99)	-34.36	0.05
Nephrology care with pre-dialysis KDE	1.08 (0.98, 1.17)	1.10 (0.95, 1.24)	1.10 (0.97, 1.23)	1.12 (0.92, 1.32)	0.98 (0.95, 1.01)	0.98 (0.93, 1.02)	-27.13	0.10
Switch to AVF/AVG among Hispanic Individuals with Incident Maturing AVF/AVG and CVC								
Any Nephrology Care	0.98 (0.79, 1.17)	1.28 (0.90, 1.66)	1.01 (0.81, 1.22)	1.34 (0.93, 1.75)	0.97 (0.95, 0.99)	0.96 (0.94, 0.98)	-20.02	0.04
More than 6-months Nephrology Care	0.92 (0.72, 1.11)	1.23 (0.82, 1.64)	0.95 (0.74, 1.16)	1.30 (0.85, 1.75)	0.96 (0.94, 0.99)	0.94 (0.92, 0.97)	-32.12	0.14
Nephrology care with pre-dialysis KDE	0.93 (0.77, 1.10)	1.02 (0.78, 1.27)	0.94 (0.77, 1.11)	1.02 (0.78, 1.27)	0.99 (0.96, 1.02)	1.00 (0.98, 1.02)	1.05	0.98

eTable4 notes: KDE: KRT-directed kidney disease education; AVF: Arteriovenous Fistula; AVG: Arteriovenous Graft; CVC: Central Venous Catheter; Maturing accesses refer to an AVF or AVG in situ but not usable on first dialysis; aOR: adjusted odds ratio; 95%CI: 95% confidence interval.

Switch to AVF/AVG refers to those with incident CVC with or without maturing Arteriovenous access, who are able to use AVF or AVG during the first year on dialysis.

The procedure estimates mediation effects from a set of relationships between an outcome variable (Switching to AVF/AVG from CVC), a treatment variable (Race/Ethnicity), a mediator variable (Nephrology care, more than 6 months nephrology care, KDE) and a set of background covariates (age, sex, diabetes, congestive heart failure, coronary artery disease, body mass index, facility profit status, low-income subsidy indicator, rurality.).
The Natural Direct Effect refers to the direct effect of Race/Ethnicity on vascular access In absence of the Mediator. The Natural Indirect Effect refers to the effect of Race/Ethnicity on switching to AVF/AVG through the mediator. The Total Effect = (Natural Direct Effect) x (Natural Indirect Effect). The adjusted mediation percentage is the percent of the adjusted Total Effect which is attributable to the Natural Indirect Effect.

© 2025 Pramod S et al. *JAMA Network Open*.
