# Supplementary material for: Predialysis Nephrology Care Disparities and Incident Vascular Access Among Hispanic Individuals
Source: JAMA Netw Open. 2025 Sep 5;8(9):e2530972. doi: 10.1001/jamanetworkopen.2025.30972 (PMC12413648; doi:10.1001/jamanetworkopen.2025.30972)
Supplement: Supplement 2. — Data Sharing Statement [file jamanetwopen-e2530972-s002.pdf]

Data Sharing Statement

Pramod. Predialysis Nephrology Care Disparities and Incident Vascular Access Among Hispanic Individuals. *JAMA Netw Open*. Published September 05, 2025. doi:10.1001/jamanetworkopen.2025.30972

Data
Data available: No

Additional Information
Explanation for why data not available: All presented data are avalialble through USRDS.
